# Louisville Summer School of Medicine

**Published:** 1846-09

**Authors:** 


### LOUISVILLE SUMMER SCHOOL.

The lectures in the Louisville Summer School of Medicine will commence on the first Monday in September and continue to the end of October. During the latter month the dissecting rooms will be open under the direction of the Demonstrator of Anatomy, Dr. Bayless. Sudents of medicine are earnestly advised to come on and embrace the admirable opportunity afforded by this arrangement for making themselves practical anatomists. Dr. Bayless conducts his department with singular success; his tables are always well supplied, and he has always large classes. No scarcity of material has ever been complained of by those who have taken his ticket, and the policy of the school is to encourage dissections. By an ordinance lately adopted, every student must take the dissecting ticket one season before he can present himself for his degree. The effect of the fall course of lectures and of throwing open the anatomical rooms to students in October, is to lengthen the lecture term more than a month. Students will see the advantage of this.

